# Twelve Chinese herbal preparations for the treatment of depression or depressive symptoms in cancer patients: a systematic review and meta-analysis of randomized controlled trials

**DOI:** 10.1186/s12906-019-2441-8

**Published:** 2019-01-23

**Authors:** Menglin Li, Zijie Chen, Zhenzhu Liu, Ning Zhang, Jintao Liu, Huiru Wang, Weiguang Wang, Yan Liang, Jingwen Chen, Zhe Liu, Yongle Li, Shuangqing Zhai

**Affiliations:** 1 School of Chinese Medicine, Beijing University of Chinese Medicine, Beijing, 100029 China; 2 College of Basic Medicine, Hubei University of Chinese Medicine, Hubei, 430065 China; 3 College of Traditional Chinese Medicine, Inner Mongolia Medical University, Hohhot, 010110 China

**Keywords:** Chinese herbal medicine, Depression, Depressive symptoms, Cancer

## Abstract

**Background:**

Patients with cancer are vulnerable to depression or other depressive conditions. The aim of this paper was to evaluate the efficacy and safety of Chinese herbal medicine (CHM) for the treatment of depression or depressive symptoms in cancer patients.

**Methods:**

CENTRAL, MEDLINE, EMBASE, PsycINFO, CNKI, VIP, SinoMed, and online clinical trial registry websites were searched for relevant RCTs until May 2017. The methodological quality of each included study was assessed with the “risk of bias” tool. Review Manager 5.3.5 was used to analyze the data.

**Results:**

We identified 18 RCTs that included data from 1441 participants. Twelve different types of Chinese herbal preparations were investigated by these studies, and they showed a better therapeutic effect in most comparisons when measured in terms of depression rating scale scores, with SMDs (95% CI) of − 2.30 (− 3.54, − 1.05) (CHM versus no treatment), − 0.61 (− 1.03, − 0.18) (CHM versus antidepressants), and − 0.55 (− 1.07, − 0.02) (CHM plus psychological treatments versus psychological treatments), or when measured in terms of treatment response rate, with RRs (95% CI) of 1.65 (1.19, 2.29) (CHM versus no treatment), 1.15 (1.03, 1.28) (CHM versus psychological treatments), 1.32 (1.07, 1.63) (CHM plus antidepressants versus antidepressants), and 1.70 (1.02, 2.85) (CHM plus psychological treatments versus psychological treatments). Compared with antidepressants, these CHMs showed borderline superiority for improving the response rate, with an RR (95% CI) of 1.08 (0.93, 1.26). Subgroup analysis based on psychiatric diagnosis (depression versus depressive symptoms) did not modify the direction of these estimates and neither could it explain the high level of heterogeneity. Patients in the CHM group experienced fewer adverse events of cardiac toxicity (*P* = 0.02), functional gastrointestinal disorders (*P* = 0.008), sleep disturbances (P = 0.02), blurred vision (P = 0.02) and fatigue (*P* = 0.03) than the patients in the no treatment group or the antidepressants group.

**Conclusions:**

According to the investigation of the twelve herbal preparations, the CHM intervention appears to alleviate depressive symptoms in cancer patients, either alone or combined with antidepressants or psychological treatments. However, a high risk of bias and high heterogeneity made the mean estimates uncertain. Well-designed trials with comprehensive and transparent reporting are warranted in the future.

**Electronic supplementary material:**

The online version of this article (10.1186/s12906-019-2441-8) contains supplementary material, which is available to authorized users.

## Background

Survival after cancer has improved over the past three decades [1], and approximately 67% of patients live for at least 5 years after diagnosis [2]. Despite these gains in cancer treatment, long-term behavioral co-morbidities, such as depression or other depressive conditions, are prominent. Reactions to the cancer diagnosis, unpleasant symptoms related to cancer, concerns about disease progression, and the physiological effects of certain anticancer treatments can all increase patients’ susceptibility to depressive symptoms. According to a meta-analysis of 94 interview-based studies, the estimated prevalence of depression and other depressive conditions was 16.5% in palliative care settings and 16.3% in non-palliative care settings [3].

Most cancer patients with depression are neither diagnosed nor treated [4], although the presence of depression is associated with negative outcomes, such as a substantial decrease in quality of life [5] and an increased mortality rate for the cancer itself [6–8]. Consequently, reports from a National Institutes of Health (NIH) panel [9] and the Institute of Medicine (IOM) [10] and clinical guidelines from the National Comprehensive Cancer Network (NCCN) [11] and the National Institute for Health and Care Excellence (NICE) [12] have recommended screening for psychological distress, including depression, as part of standard supportive and palliative cancer care.

The treatment of depression can mainly be divided into pharmacological and psychological interventions. Although both types of interventions have been shown to be efficacious, first-line recommendations for the treatment of depression in cancer patients are difficult to determine from current evidence [13, 14]. Conventional pharmacological treatments include selective serotonin reuptake inhibitors (SSRIs), serotonin and noradrenaline reuptake inhibitors (SNRIs), agomelatine, bupropion, mirtazapine and vortioxetine [15]. However, patients taking antidepressant drugs often experience high relapse rates and a variety of side effects, such as nausea, headaches, somnolence, dry mouth and male sexual dysfunction [15]. Moreover, some potentially harmful drug-drug interactions have been proven between antidepressants and cancer chemotherapeutic agents [16–18] and anti-emetics [19]. Recently, new treatment strategies, such as drugs that target the neuroendocrine system, cytokines or pro-inflammatory signaling, have been generated to address the aberrant pathways linking depression and cancer [20]. However, the risk of severe gastrointestinal or cardiovascular events hinders the application of these new drugs in clinical practice. Psychological interventions also include a wide range of specific therapies, such as cognitive behavioral therapy (CBT), interpersonal therapy (IPT), and behavioral activation (BA) [21]. However, due to their time-intensive nature, inadequate access to skilled providers, high costs, and requirements of patients’ participation and motivation, the application of psychosocial interventions is limited. Therefore, Chinese herbal medicine (CHM), a cost-effective and lower-toxicity form of alternative medicine, has gained increasing attention as a therapeutic method for treating depression in cancer patients.

CHM generates therapeutic effects through multiple pathways, such as monoamine transmission system enhancement [22, 23], HPA axis activity down-regulation [24], and anti-inflammation and immunity regulation [25], and it views health and disease through a holistic perspective incorporating body, mind, and spirit. Although clinical studies regarding the use of CHM for depression or depressive symptoms in cancer patients have been conducted and have reported potentially positive results [26–28], no systematic review has been performed to justify the clinical use of CHM for this purpose. Therefore, this review aimed to assess the effectiveness and safety of CHM for the treatment of depression or depressive symptoms in cancer patients.

## Methods

The review has been prospectively registered in PROSPERO. The registration number is CRD42017063831.

### Eligibility criteria

We included RCTs in which cancer patients also fulfilled the diagnostic criteria for depression (major depressive disorder, MDD) as stated by a well-established diagnostic system, such as the Diagnostic and Statistical Manual of Mental Disorders-IV (DSM-IV) [29], DSM-V [30], International Classification of Diseases 10 (ICD-10) [31], Chinese classification of mental disorders-2R (CCMD-2R) [32] and CCMD-3 [33], or presented with depressive symptoms as indicated by specialized depression rating scales (e.g., the Hamilton Rating Scale for Depression (HAMD) [34], the Zung Self-Rating Depression Scale (ZSDS) [35], the Hospital Anxiety and Depression Scale (HADS) [36], the Beck Depression Inventory (BDI) [37], or the Center for Epidemiologic Studies–Depression Scale (CES-D) [38]) with evidence of adequate validity and reliability.

The CHM interventions included single herbs, herbal products extracted from natural herbs, herbal decoction, or Chinese proprietary medicines approved by the China State Food and Drug Administration. The control interventions included no treatment, placebo or conventional interventions used with the intention of alleviating depressive symptoms. Therapies combining CHM and other interventions were also included and compared with other interventions alone.

The outcome measurements included group mean scores on rating scales for depression, the response rate for depression, group mean scores on rating scales for quality of life, the response rate for quality of life and the incidence of adverse events. Clinical response was defined as achieving a reduction of at least 50% on the validated rating scale or meeting other criteria pre-specified in the original literature. We gave preference to the endpoints stated in the original trials if the participants were measured at different time points.

### Search strategy

We searched the following bibliographic electronic databases to identify relevant studies for this review: CENTRAL, MEDLINE, EMBASE, PsycINFO, the Chinese National Knowledge Infrastructure Databases (CNKI), Chinese VIP information (VIP), the Chinese Biomedical Database Web (SinoMed), and the Wanfang Database. The search duration was from the inception of the databases to May 2017. Ongoing registered clinical trials were searched through the World Health Organization (WHO) International Clinical Trial Registry Platform (ICTRP) portal, the website of the International Clinical Trial Registry of the U.S. NIH (http://clinicaltrials.gov/) and the website of the Chinese clinical trial registry (http://www.chictr.org.cn/). Language, publication year and publication status were not limited. The reference lists of all eligible articles were also obtained.

The following search terms were used individually or combined: “Chinese medicine”, “traditional medicine”, “herbal medicine”, “Oriental medicine”, “phytomedicine”, “botanical”, “herb”, “plant”, “neoplasm”, “cancer”, “tumour”, “carcinoma”, “malignant”, “metastasis”, “adenocarcinoma”, “sarcoma”, “lymphoma”, “choriocarcinoma”, “leukaemia”, “teratoma”, “melanoma”, “blastoma”, “glioma”, “chordoma”, “mesothelioma”, “depression”, “affective disorder”, “depressive disorder”, “mood disorder”, “reactive disorder”, “dysthymic disorder”, “adjustment disorder”, “mental health”, “melancholia”, “dysthymia”, “meta-analysis”, “blind”, “placebo” and “random”. The search strategy is listed in Additional file 1.

### Study selection and data extraction

Two reviewers (MLL, ZZL) independently assessed the titles, abstracts and keywords of every record retrieved. The full texts of all potentially relevant articles were investigated. Disagreements were resolved by discussion between the two review authors and, if necessary, with a third review author (ZJC).

Data were independently extracted from the included trials by two review authors (MLL, NZ) and were entered into the structured characteristics table. For each trial, we extracted the publication year, study sample size, diagnosis criteria, methodological details, demographic characteristics, cancer information, details regarding the herbal medicine and control interventions, follow-up duration, attrition rates, outcomes, adverse events and funding. We resolved any differences in opinion through consultation with a third person (ZJC).

### Assessment of risk of bias

Each included trial was independently assessed for risk of bias using the criteria described in the Cochrane Handbook version 5.1.0 [39]. The assessments were performed by the authors, with any disagreements resolved by discussion with a third party. We assessed the following domains for each study: random sequence generation, allocation concealment, blinding of participants and personnel, blinding of outcome assessments, incomplete outcome data, selective reporting, and other sources of bias. The quality of each trial was classified as low, unclear, or high risk of bias. Given that many of the studies might have been conducted without registration, we checked their Methods and Results sections to assess reporting bias. In addition, we contacted the authors of assessed trials for clarification if necessary.

### Data analysis

RevMan 5.3.5 was used to analyze the results of the studies. If a sufficient number of clinically similar studies were available, we pooled their results in the meta-analysis. Continuous data are reported as the mean differences (MDs) or standardized mean differences (SMDs), whereas dichotomous data are reported as relative risks (RRs) with 95% confidence intervals (CIs). In addition, we assessed the selected trials for the type of intervention used and grouped the trials accordingly. Since differences in cancer stage, gender and psychiatric diagnosis (i.e. the difference between depression and depressive symptoms) may influence treatment efficacy or be an important reason for heterogeneity in the interventions, subgroup analyses were conducted if a sufficient number of trials were included. We investigated heterogeneity between trials using the I^2^ statistic [40, 41] and by visual inspection of the forest plots. Funnel plots were generated to detect publication bias when more than ten trials were identified [39].

## Results

### Study selection

**Fig. 1:**
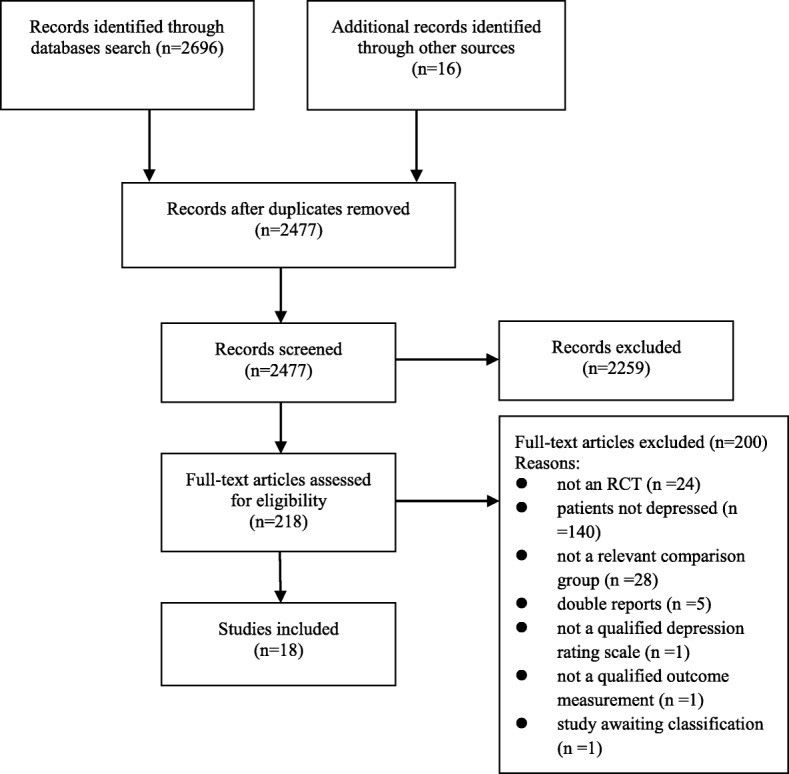
Flow diagram

We identified 2696 references through the search strategies. After removing duplicates and checking the titles and abstracts, we retrieved 218 full-text articles. Of these, 18 studies fulfilled the inclusion criteria and were included in this review [26–28, 42–56]. Additionally, one trial (ClinicalTrials.gov Identifier: NCT00066859) was assigned to the “awaiting classification” list based on its abstract, buts its authors have not yet replied to our request for data. Details are displayed in Fig. 1.

### Description of studies

In total, 1441 adult patients were randomized in 18 trials. Fifteen studies [26–28, 42, 43, 45, 46, 49–56] enrolled patients with a formal diagnosis of depression, while three studies recruited people with depressive symptoms according to their cut-off scores on standardized rating scales [44, 47, 48]. In total, the included trials tested twelve different types of Chinese herbal preparations (eleven were herbal decoction and one was a Chinese patent medicine). Among these, Xiao Yao decoction or its modifications were the most frequently used, accounting for 22.2% of the total number of formulae tested. The characteristics of these trials are presented in Table 1, and the specific compositions of these Chinese herbal preparations are shown in Additional file 2.

**Table 1:** Characteristics of the included randomized controlled trials

Author, year	Sample size R/A*	Age (years)	Women (%)	Diagnostic criteria	Cancer	Intervention T/C*	Anticancer therapies	Practice mode	Outcomes
Chen et al. [26]	120/120	T: 64.1 ± 6.3 C: 60.0 ± 9.1	70.0	Depression: CCMD-3	Lung and breast cancers; any stage	T: Xuefuzhuyu decoction,1 dose/d; C: no treatment	Y	IT*	1, 2
Liu et al. [42]	38/38	T: 58.9 ± 4.8 C: 59.6 ± 4.5	55.3	Depression: CCMD-3, SDS > 50, HAMD-24 > 7	Lung cancer; stage IIIB to IV	T: Yangfeixiaojijieyu decoction, 1 dose/d; C: no treatment	Y	NIT*	1, 2, 4
Ma et al. [43]	81/81	T: 53.9 ± 10.7 C: 56.0 ± 11.9	60.5	Depression: CCMD-2R, HAMD-17 > 18	Gastric, lung, esophageal, breast, liver and other cancers; any stage	T: Danzhixiaoyao decoction,1 dose/d, 8 weeks; C: no treatment	Y	IT	1, 2
Meng et al. [44]	78/78	median T: 61.2 C: 62.2	41.0	Depressive symptoms: SDS > 53	Esophageal cancer; any stage	T: Chaihushugan decoction alternatively combined with Tongyou decoction; C: no treatment	Y	NIT	1
Meng et al. [45]	100/100	T: 68.7 ± 1.4 C: 69.7 ± 1.7	31.0	Depression: CCMD-3, SDS > 50, HAMD-24 > 20	Breast, gastric, colorectal and lung cancers; any stage	T: Ganmaidazao decoction, 15 d; C: no treatment	Y	IT	1, 3
Sun et al. [46]	64/64	T: 46.0 ± 11.7 C: 46.3 ± 12.3	100.0	Depression: ICD-10, DSM, CCMD-3	Breast cancer; any stage	T: Xiaoyao decoction, 1 dose/d, 6 weeks; C: no treatment	Y	IT	1, 4, 5
Wu et al. [47]	82/77	T: 53.4 ± 16.8 C: 52.6 ± 15.7	41.5	Depressive symptoms: HAMD	Gastric cancer; Stage III to IV	T: Shuganjieyu capsule, 0.72 g, bid, 6 weeks; C: no treatment	Y	NIT	1, 2, 5
Fang et al. [48]	90/90	T: 42.3 ± 18.1 C: 47.6 ± 16.9	54.4	Depressive symptoms: SDS > 50, HAMD-17 > 14	Lung, breast, colorectal, liver and other cancers; any stage	T: Chaihushugan decoction, 1 dose/d, 6 weeks; C: fluoxetine, 20 mg qd for 2 weeks, then 20–40 mg qd for 4 weeks	Y	NIT	1, 2, 5
Fu et al. [49]	26/26	T: 41–65 C: 39–68	42.3	Depression: CCMD-3, 14 ≤ HAMD-17 ≤ 24	Breast, esophageal, rectal, nasopharynx, gastric and lung cancers; any stage	T: Suanzaorenjialongmu decoction, 1 dose/d, 6 weeks; C: fluoxetine, 20 mg qd for 2 weeks, then 20–40 mg qd for 4 weeks	NR	NIT	1, 2, 5
Jin et al. [50]	82/82	T: 23.8 ± 7.7 C: 22.9 ± 6.9	NR	Depression: CCMD-3	Breast cancer; stage I to III	T: Xiaoyao decoction, 1 dose/d, 10 weeks; C: alprazolam,0.4 mg bid, 10 weeks	NR	NIT	1, 5
Liu et al. [27]	60/60	T: 51.4 ± 8.7 C: 51.3 ± 6.6	100.0	Depression: CCMD-3, SDS > 50	Breast cancer; stage I to IIIC	T: Xiaoyao decoction, 1 dose/d, 6 weeks; C: flupenthixol and melitracen, 1 tablet bid, 6 week	NR	IT	1, 2, 5
Ma et al. [51]	60/60	18–80	46.7	Depression: DSM-IV	Breast, esophageal, colorectal, gastric, lung, ovarian, prostatic and liver cancers; stage II to IV	T: Shuganjieyuhuaji decoction, 1 dose/d, 6 weeks; C: fluoxetine 20 mg qd, 6 weeks	NR	NIT	1, 2, 3
Tian et al. [52]	120/120	30–70	NR	Depression: CCMD-3, HAMD≥16	Breast, pancreatic, esophageal, lung and gastric cancers; any stage	T: Chaihujialonggumuli decoction,1 dose/d + PT, 6 weeks; C: fluoxetine 20 mg qd + PT, 6 weeks	NR	NIT	1, 2
Zhang et al. [53]	98/98	T: 58.0 ± 8.5 C: 57.0 ± 9.4	54.1	Depression: CCMD-3, HAMD> 20	Breast, colorectal, esophageal, lung and gastric cancers; any stage	T: Ganmaidazao decoction, 1 dose/d, 8 weeks; C: flupenthixol and melitracen 10.5 mg bid, 8 weeks	Y	NIT	1, 2, 5
Zheng et al. [54]	126/126	T: 48 ± 16 C: 49 ± 15	43.7	Depression: “diagnosed by psychiatrist”	NR; any stage	T: Chaihushugan decoction, 1 dose/d, 30 days; C: PT	NR	NIT	2
Jia et al. [55]	78/78	T: 53.0 ± 3.6 C:53.0 ± 4.2	78.2	Depression: CCMD-2R	Lymphoma, osteosarcoma, breast, liver, endometrial, gastrointestinal, ovarian and renal cancers; any stage	T: oral Chinese herbal medicine decoction, 1 dose/d, 4 weeks; C: fluoxetine 20 mg qd 4 weeks	NR	IT	2
Dai et al. [28]	80/80	T: 64.6 ± 10.8 C: 59.7 ± 9.8	45.0	Depression: CCMD, HAMD> 18	Gastric cancer; any stage	T: Banxiahoupo decoction+liujunzi decoction, 1 dose/d; C: PT	NR	NIT	2
Xu et al. [56]	58/58	T: 63.9 ± 10.8 C: 58.9 ± 9.6	69.0	Depression: CCMD, HAMD-17 > 17	Gastric cancer; any stage	T: Banxiahoupo decoction+liujunzi decoction, 1 dose/d; C: PT	NR	NIT	1, 2

*CHM* Chinese herbal medicine, *PT* psychological treatments, *NR* not reported, *IT* individualized treatments, *NIT* non-individualized treatments, *HAMD* Hamilton Depression Scale, *SDS* Self-Rating Depression Scale, *EORTC QLQ C*-30 European Organization for Research on Treatment of Cancer Quality of Life Questionnaire-30, *CCMD* Chinese Classification of Mental Disorders, *DSM* Diagnostic and Statistical Manual of Mental Disorders, *ICD* International Classification of Diseases

*R/A: randomized number/analyzed number

*T/C: interventions other than the anticancer treatment of the treatment group and the control group, respectively

Outcomes: 1) group mean scores on rating scales for depression; 2) response rate for depression; 3) group mean scores on rating scales for quality of life; 4) response rate for quality of life; 5) the incidence of adverse events

The included studies examined the following comparisons: CHM versus no treatment (seven trials [26, 42–47]), CHM versus antidepressants (seven trials [27, 48–53]), CHM versus psychological treatments (one trial [54]), CHM plus antidepressants versus antidepressants (one trial [55]), and CHM plus psychological treatments versus psychological treatments (two trials [28, 56]).

### Methodological quality

**Fig. 2:**
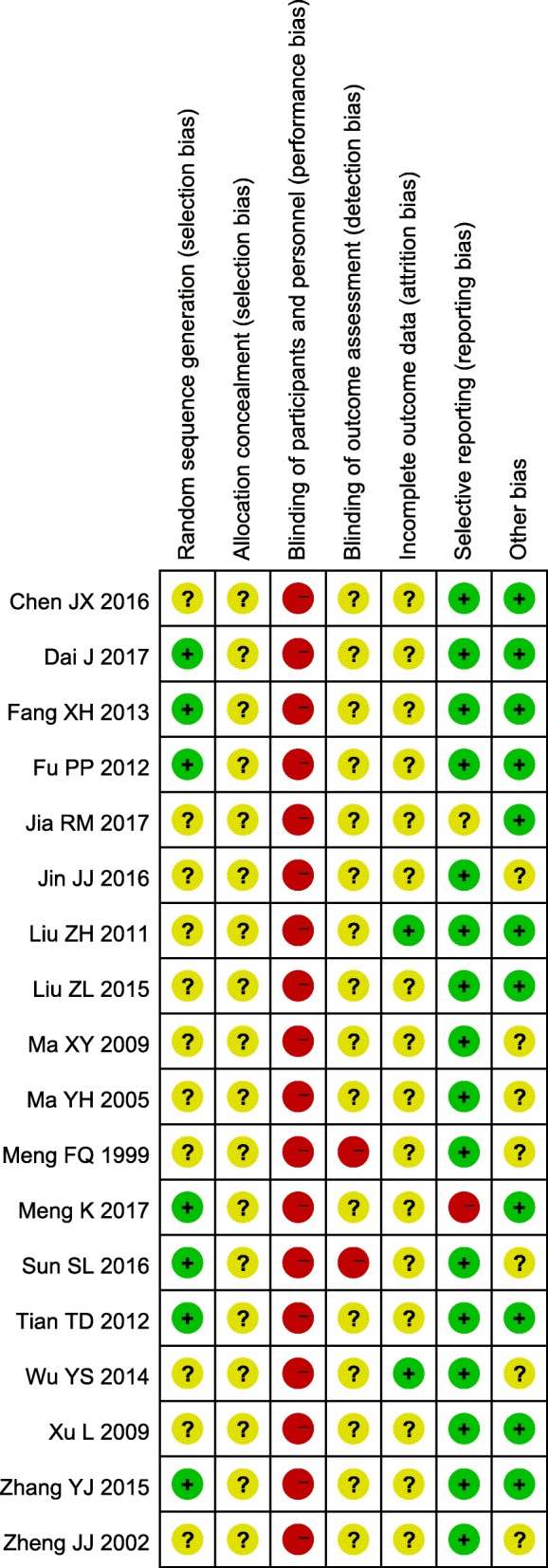
Risk of bias summary

All the included trials were determined to be of generally poor methodological quality. Eleven out of the 18 studies did not provide sufficient information about the randomization process, one trial [28] used a computer to generate a random sequence, and six [45, 46, 48, 49, 52, 53] used a random number table for randomization. No studies described allocation concealment. Blinding of the participants or study personnel was not performed in any of the included trials. Self-report questionnaires were applied in two trials [44, 46], and other-report questionnaires were applied in the remaining 16 trials [26–28, 42, 43, 45, 47–56], in which the blinding of assessors was not mentioned. Two trials [27, 47] reported missing outcome data: one indicated that no patients withdrew from one study [27], and the other reported that 6% of the participants dropped out [47], with the reasons for leaving balanced between the two groups. Since we failed to find accessible protocols for any of the included trials, we chose to check the Methods and Results sections of each study to assess the reporting bias. We found a high risk of bias in only one study [45], which did not report the previously mentioned side effects. Regarding other potential sources of bias, two trials [51, 54] did not report baseline comparability, five trials [43, 44, 46, 47, 50] failed to provide an adequate description of the inclusion and exclusion criteria, and no trial performed a sample size calculation. Unfortunately, we could not obtain additional useful information by contacting the authors. The summary of each risk of bias item for each included study is shown in Fig. 2.

### Effects estimates

#### Depression: Group mean scores

**Fig. 3:**
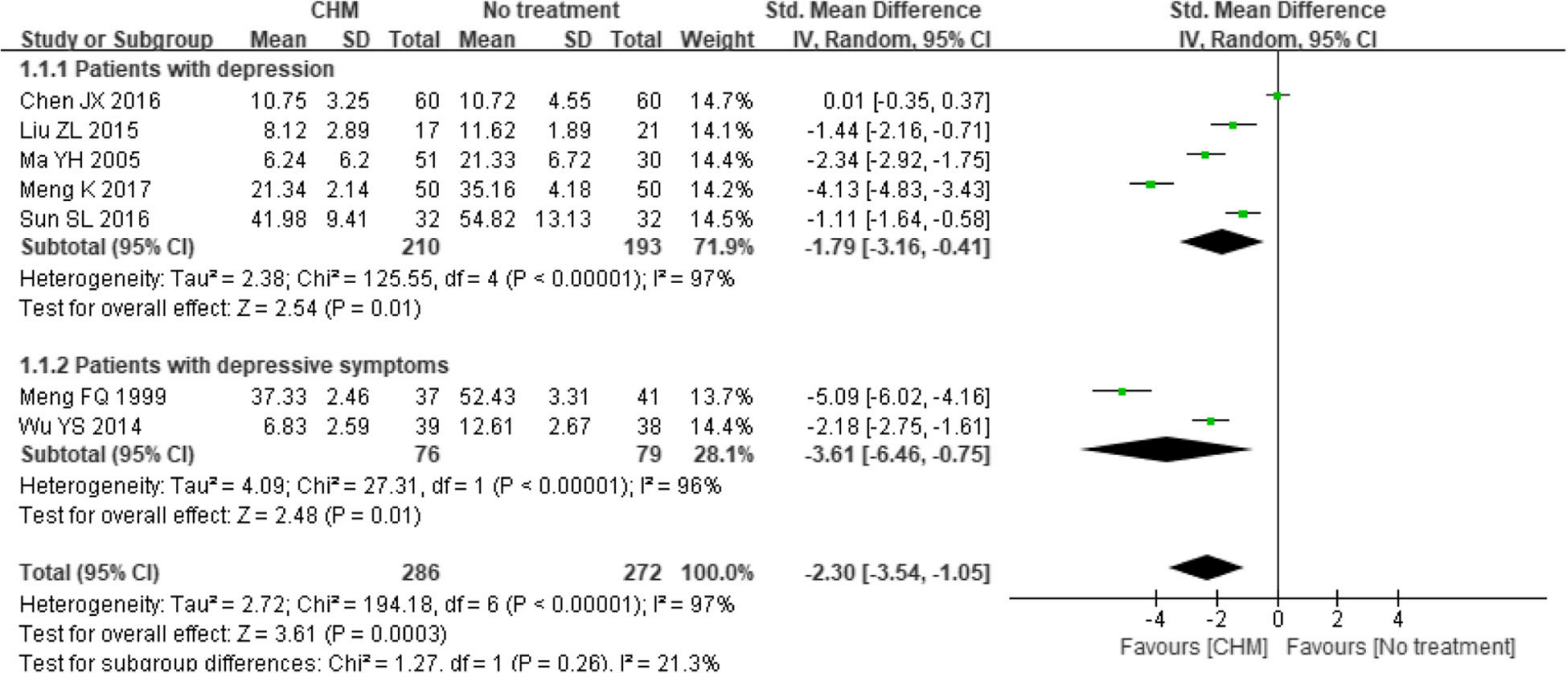
Forest plot of mean depression scores for CHM versus no treatment

The effects of CHM intervention on mean depression scores were investigated in 15 studies [26, 27, 42–53, 56]. In seven trials [26, 42–47] that compared CHM with no treatment, the CHM intervention was found to yield antidepressant effects with an SMD of − 2.30 (95% CI -3.54 to − 1.05; *P* = 0.0003). However, this effect estimate was associated with considerable heterogeneity, reflected by I^2^ = 97% (*P* < 0.00001). The direction of the estimates did not change when each of the seven trials was analyzed separately based on psychiatric diagnosis. Although the SMD was lower in the subgroup of patients with depressive symptoms (SMD -3.61, 95% CI − 6.46 to − 0.75; *P* = 0.01; I^2^ = 96%) than that in the subgroup of patients with depression (SMD -1.79, 95% CI − 3.19 to − 0.41; P = 0.01; I^2^ = 97%), no statistically significant subgroup difference could be detected (*P* = 0.26) (see Fig. 3). Subgroup analyses based on cancer stage and gender were not performed due to lack of data.

**Fig. 4:**
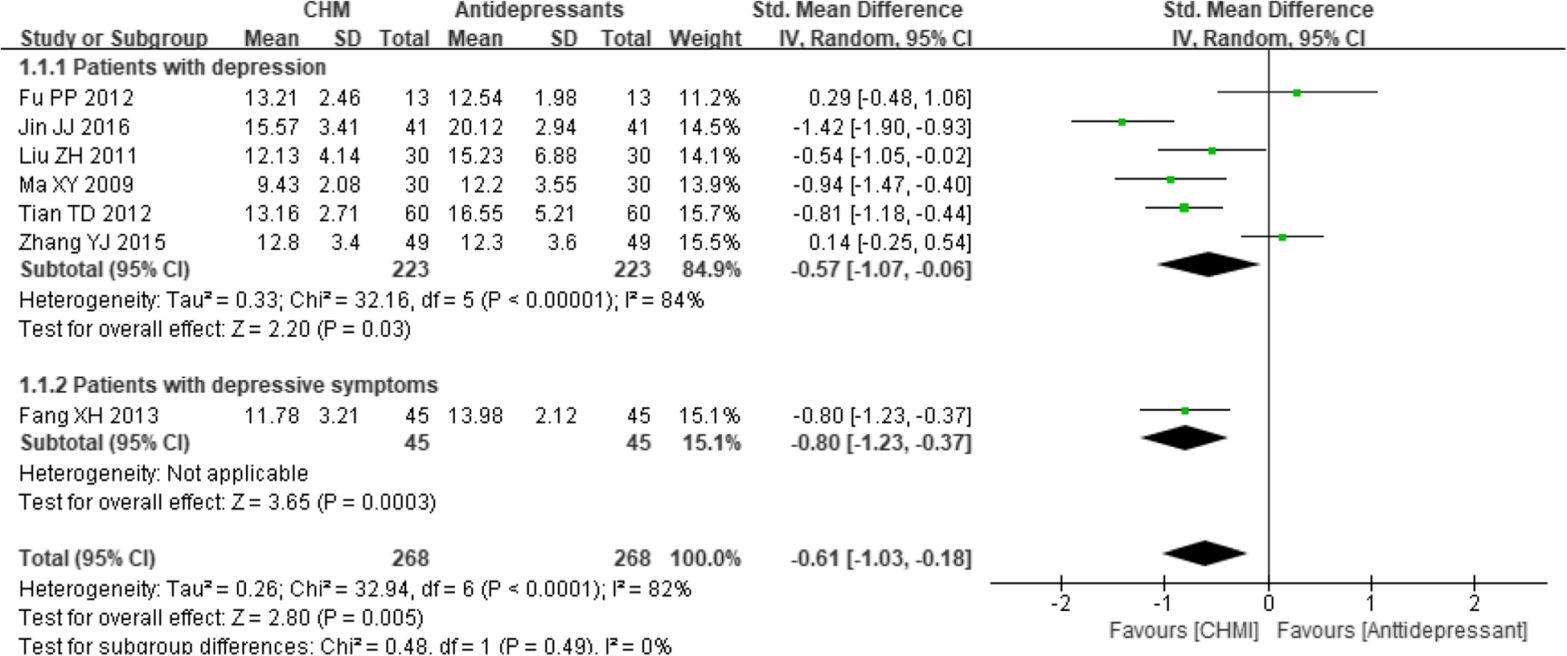
Forest plot of mean depression scores for CHM versus antidepressants

In the studies [27, 48–53] that compared CHM with antidepressants, a significant favorable effect of the CHM interventions was observed (SMD -0.61, 95% CI -1.03 to − 0.18; *P* < 0.0001), although there was considerable heterogeneity (I^2^ = 82%, *P* < 0.0001). In the subgroup analysis, the effects of the CHM intervention were superior to those of the antidepressants both in patients with depression (SMD -0.57, 95% CI -1.07 to − 0.06; *P* = 0.03; I^2^ = 84%) and in patients with depressive symptoms (SMD -0.80, 95% CI -1.23 to − 0.37; *P* = 0.0003). No statistically significant subgroup difference was detected (*P* = 0.49) (see Fig. 4). Other subgroup analyses were not performed due to lack of data.

**Fig. 5:**
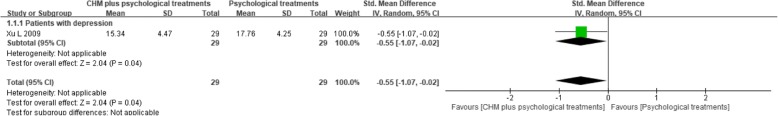
Forest plot of mean depression scores for CHM plus psychological treatments versus psychological treatments alone

Compared with psychological treatments, CHM plus psychological treatments resulted in a significant reduction in depression symptom scores [56] (MD -0.55, 95% CI -1.07 to − 0.02; *P* = 0.04) (see Fig. 5).

#### Depression: Treatment response rate

**Fig. 6:**
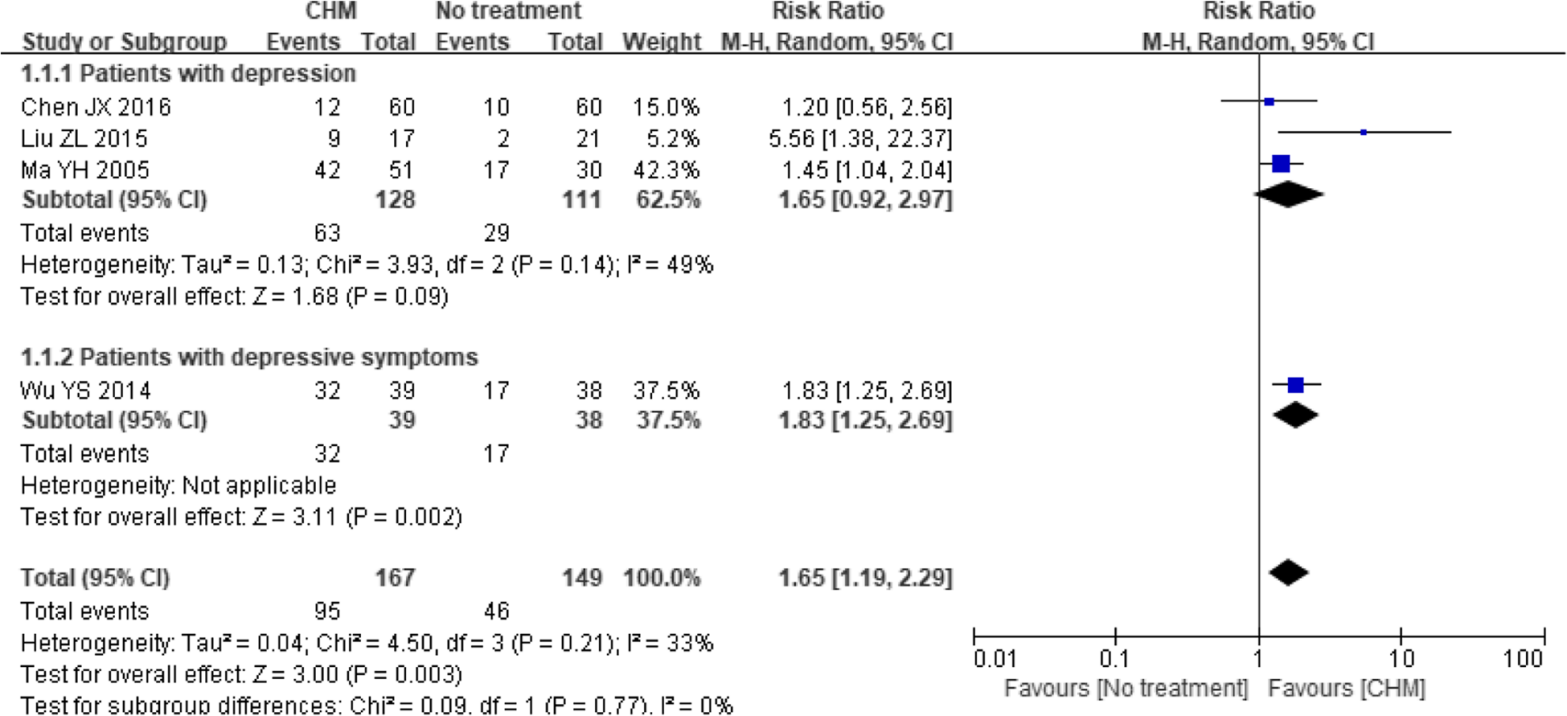
Forest plot of depression treatment response rate for CHM versus no treatment

Fourteen trials [26–28, 42, 43, 47–49, 51–56] reported the proportion of treatment responders. The effect of the CHM intervention remained significantly better than that of no treatment [26, 42, 43, 47] (RR 1.65, 95% CI 1.19 to 2.29; *P* = 0.003), and the heterogeneity among the studies was moderate (I^2^ = 33%, *P* = 0.21). However, a significant difference was demonstrated only in patients with depressive symptoms (RR 1.83, 95% CI 1.25 to 2.69; *P* = 0.002) according to the subgroup analysis (see Fig. 6).

**Fig. 7:**
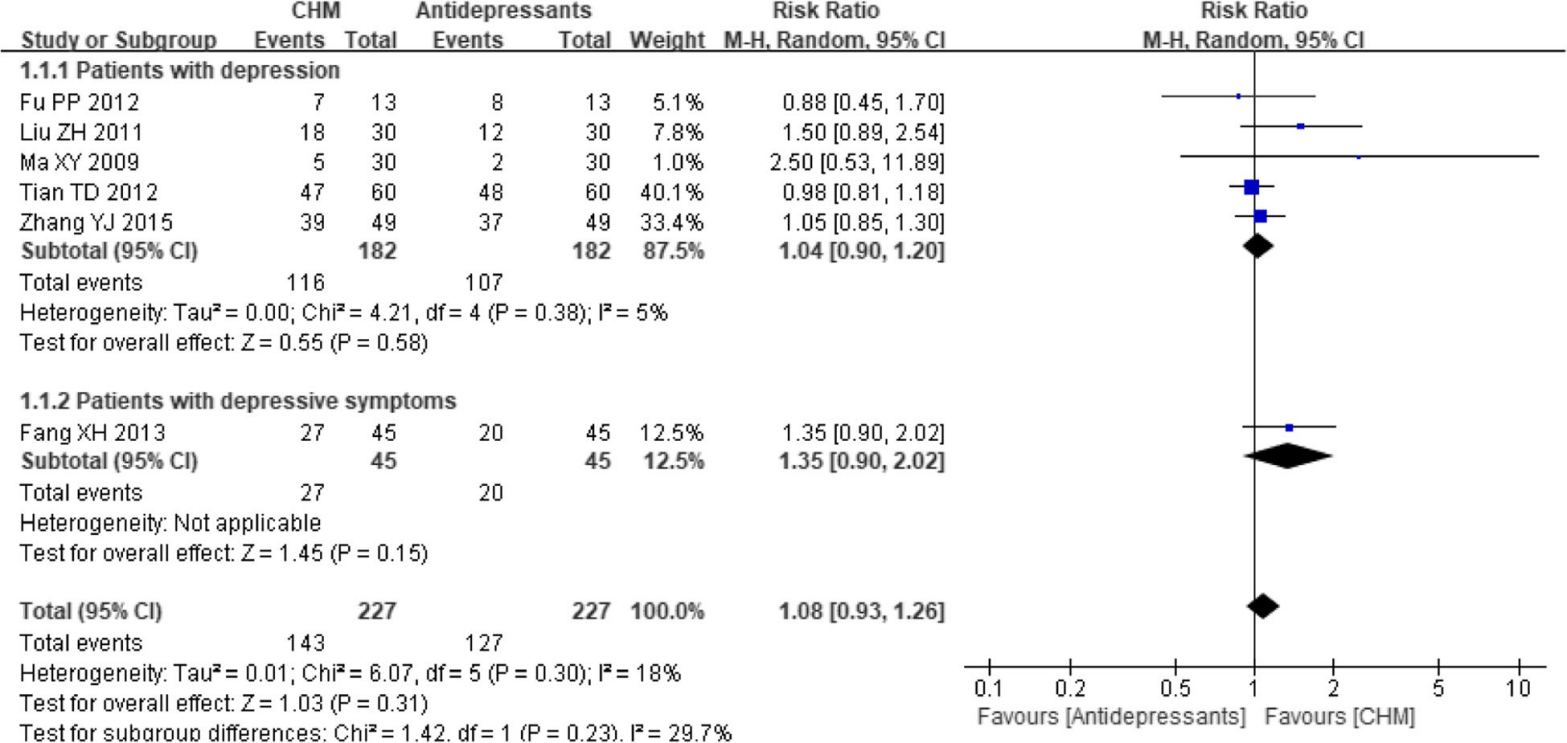
Forest plot of the depression treatment response rate for CHM versus antidepressants

When the six trials [27, 48, 49, 51–53] that compared CHM with antidepressants were analyzed, the results showed borderline significance (RR 1.08, 95% CI 0.93 to 1.26; *P* = 0.31) with little heterogeneity (I^2^ = 18%, *P* = 0.30), although a tendency towards a superior effect was noted in the CHM group. No statistically significant difference between CHM and antidepressants was observed in either subgroup (see Fig. 7).

**Fig. 8:**
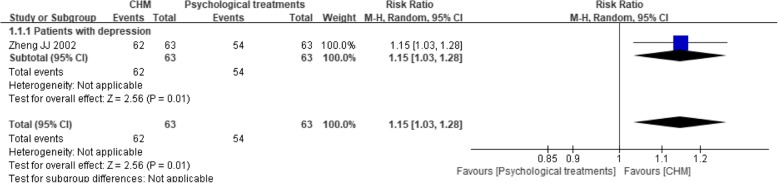
Forest plot of the depression treatment response rate for CHM versus psychological treatments

One trial [54] compared CHM to psychological treatments, and it revealed that the CHM intervention had a significant beneficial effect (RR 1.15, 95% CI 1.03 to 1.28; *P* = 0.01) (see Fig. 8).

**Fig. 9:**
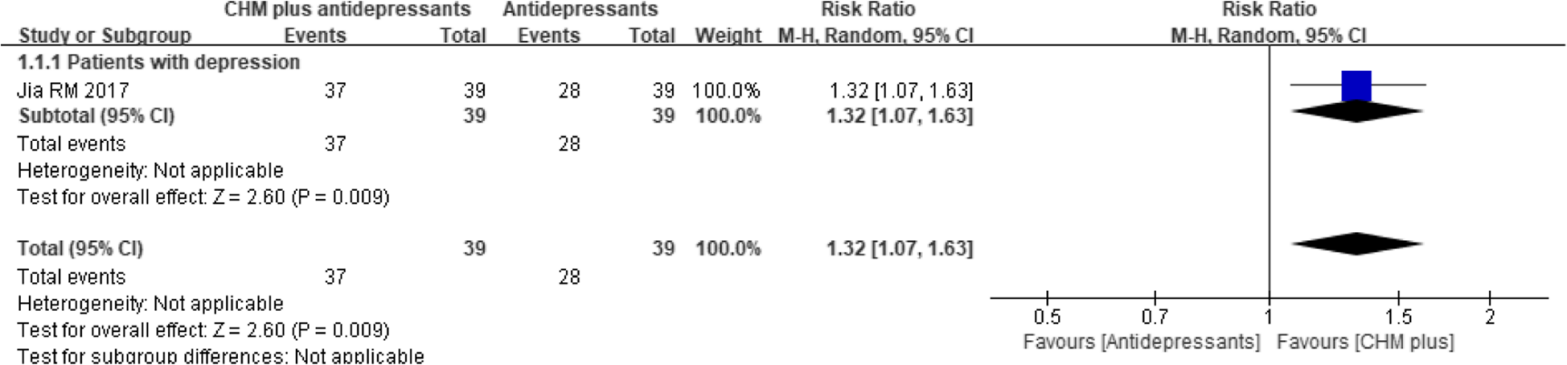
Forest plot of the depression treatment response rate for CHM plus antidepressants versus antidepressants alone

One trial [55] compared CHM plus antidepressants to antidepressants alone and found that the addition of the CHM intervention was associated with a significantly higher response rate (RR 1.32, 95% CI 1.07 to 1.63; *P* = 0.009) (see Fig. 9).

**Fig. 10:**
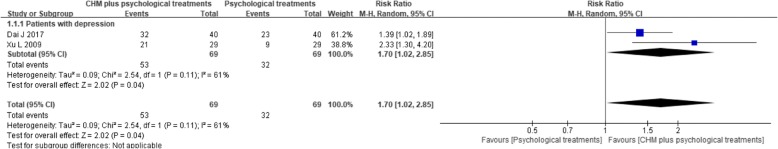
Forest plot of the depression treatment response rate for CHM plus psychological treatments versus psychological treatments alone

Additionally, two trials [28, 56] compared CHM plus psychological treatments to psychological treatments alone and found that the addition of CHM had a significantly better effect (RR 1.70, 95% CI 1.02 to 2.85; *P* = 0.04; I^2^ = 61%) (see Fig. 10).

#### Quality of life: Group mean scores

**Fig. 11:**
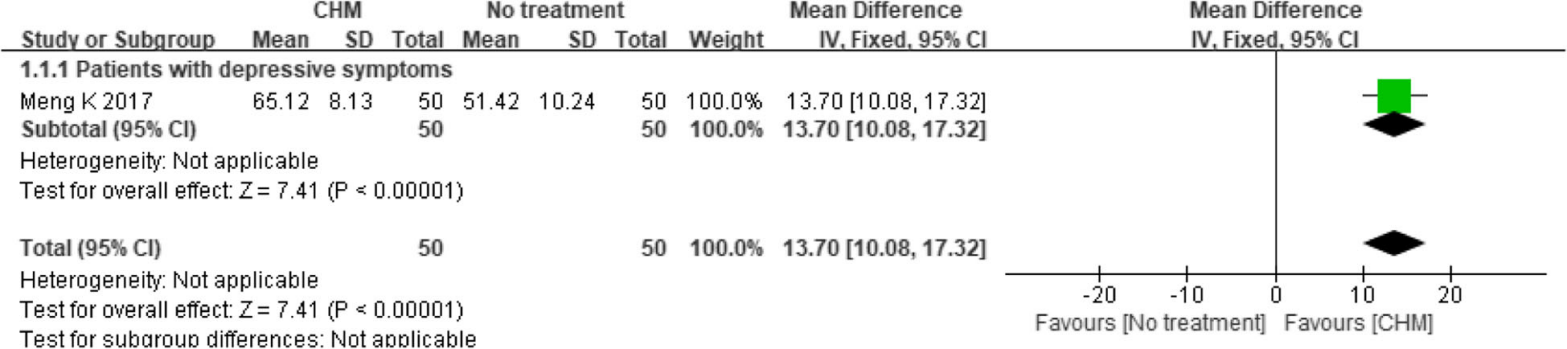
Forest plot of the group mean quality of life scores for CHM versus no treatment

**Fig. 12:**
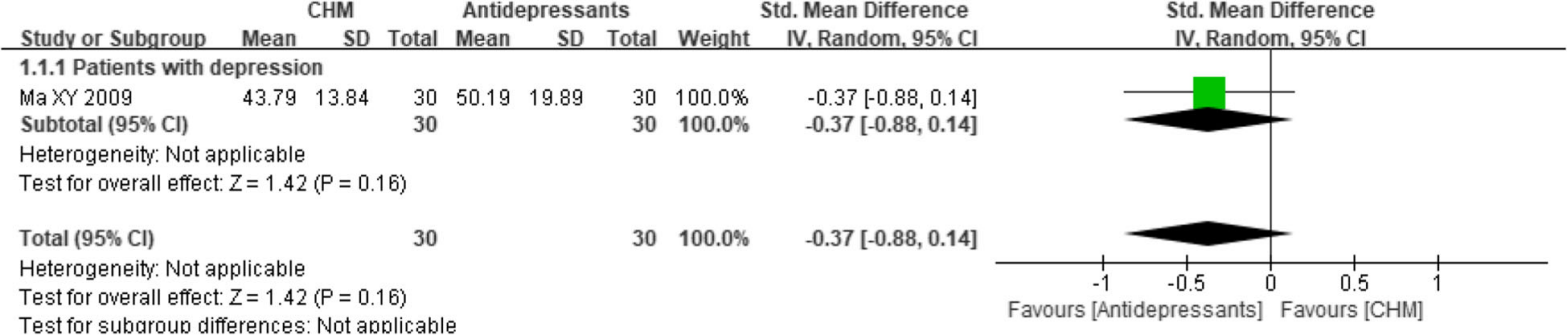
Forest plot of the group mean quality of life scores for CHM versus antidepressants

One trial [45] compared CHM with no treatment and found a significant effect of the CHM intervention (MD 13.70, 95% CI 10.08 to 17.32; *P* < 0.00001) (see Fig. 11), while another trial [51] compared CHM with antidepressants and found a non-significant difference, with an MD of − 0.37 (95% CI -0.88 to 0.14; *P* = 0.16) (see Fig. 12).

#### Quality of life: Treatment response rate

**Fig. 13:**
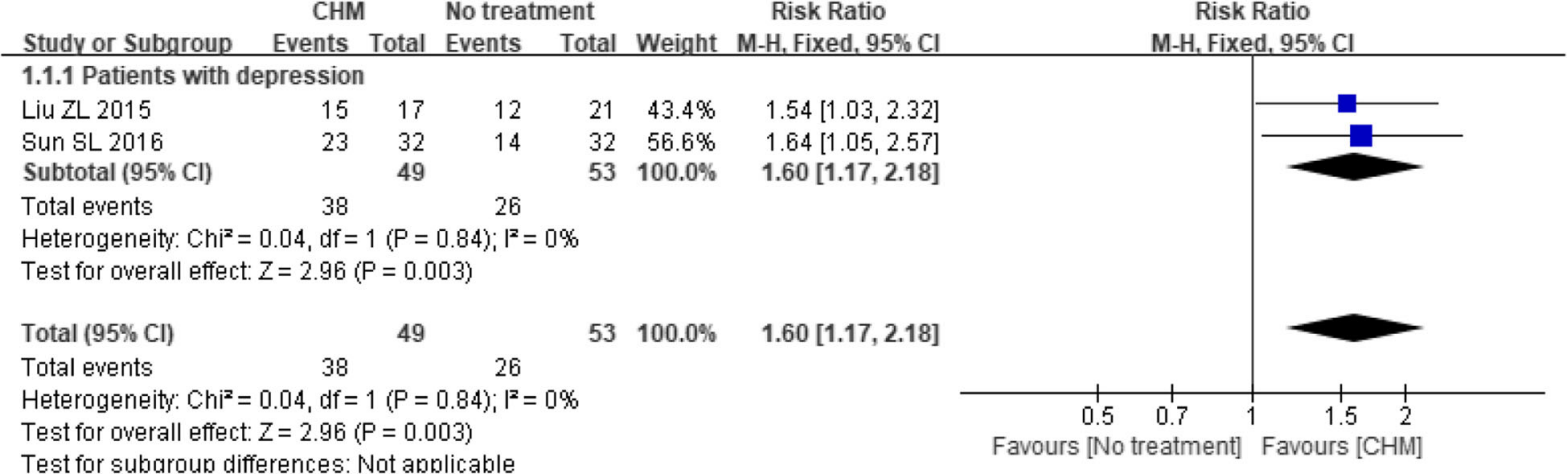
Forest plot of the treatment response rate of quality of life for CHM versus no treatment

Only two trials [42, 46] that compared CHM with no treatment measured the proportion of patients that achieved a certain degree of improvement on their quality of life rating scales. The meta-analysis indicated a beneficial effect of the CHM intervention compared with no treatment, with an estimated RR of 1.60 (95% CI 1.17 to 2.18; *P* = 0.003) and minimal between-study heterogeneity (I^2^ = 0%, *P* = 0.84) (see Fig. 13). Subgroup analyses were not performed due to the lack of suitable trials.

#### Adverse events

**Fig. 14:**
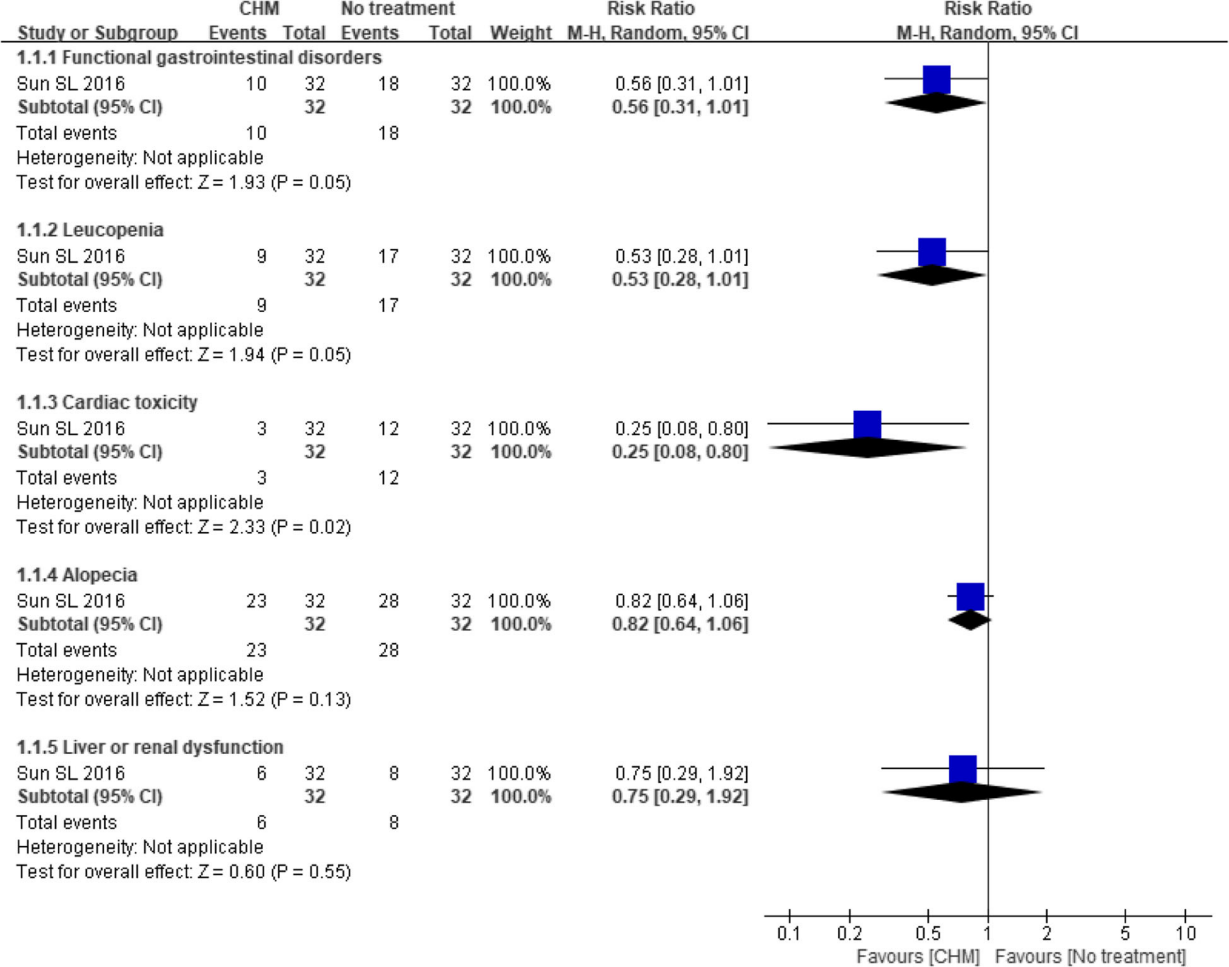
Forest plot of adverse events for CHM versus no treatment

Eight trials [27, 46–50, 52, 53] provided data regarding adverse events. Among the trials comparing CHM with no treatment, one trial [47] reported that two patients in the CHM group and two in the control group died without explaining the cause of death. Another trial [46] provided a specific description of adverse events, including nausea and vomiting, leukopenia, alopecia, cardiac toxicity and liver or renal dysfunction, that could be attributable to anticancer treatments. Although all these events occurred less frequently in the CHM group than in the control group, only the difference in the incidence of cardiac toxicity was significant (*P* = 0.02) (see Fig. 14).

**Fig. 15:**
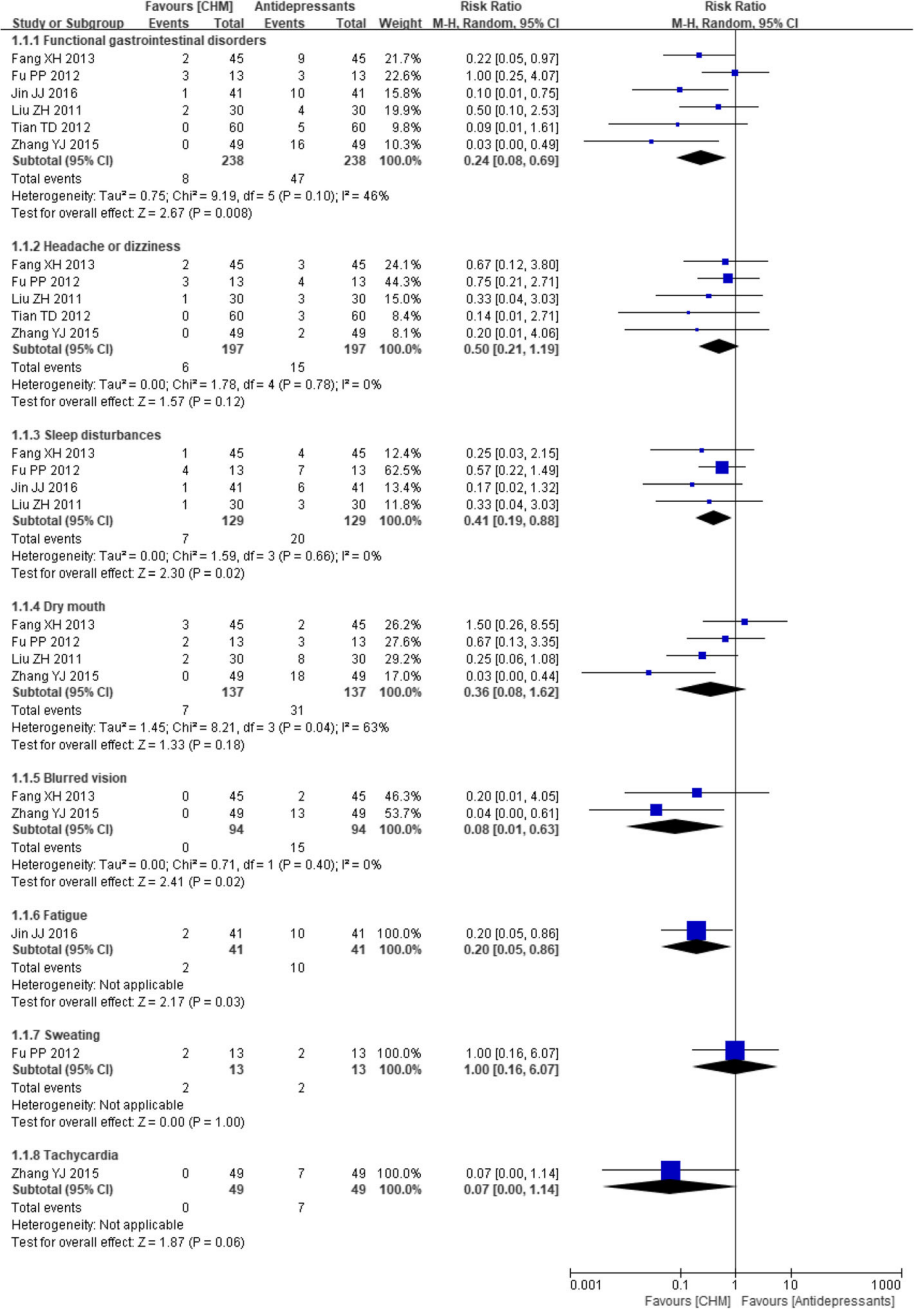
Forest plot of adverse events for CHM versus antidepressants

Of the studies comparing CHM with antidepressants, six [27, 48–50, 52, 53] measured the occurrence of adverse events. There was a statistically significant reduction in the incidence of functional gastrointestinal disorders (RR 0.24, 95% CI 0.08 to 0.69; *P* = 0.008; I^2^ = 46%), sleep disturbances (RR 0.41, 95% CI 0.19 to 0.88; *P* = 0.02; I^2^ = 0%), blurred vision (RR 0.08, 95% CI 0.01 to 0.63; *P* = 0.02; I^2^ = 0%) and fatigue (RR 0.20, 95% CI 0.05 to 0.86; *P* = 0.03) in patients taking the CHM treatment compared to those taking antidepressants. However, for the occurrence of dry mouth, headache or dizziness, sweating and tachycardia, the difference did not reach statistical significance (see Fig. 15). In addition, a subgroup analysis showed no statistically significant difference between CHM and antidepressants for preventing sleep disturbances and blurred vision in patients experiencing depressive symptoms.

The details of these effect estimates are shown in Additional file 3.

Publication bias assessment and sensitivity analysis were not performed due to the insufficient number of trials for each outcome.

## Discussion

### Summary of the main results

The present systematic review identified 18 studies involving 1441 participants. Fifteen studies provided continuous data on depression rating scale scores. The CHM treatment applied in these studies showed a significant beneficial effect and was more successful at alleviating depressive symptoms than any comparator. Subgroup analysis based on psychiatric diagnosis did not modify the direction of these estimates and neither could it explain the high level of heterogeneity. Fourteen studies reported dichotomous data regarding the response to treatment. Although the CHM intervention was more effective in most of the studies, the difference was not statistically significant when compared to the effect of antidepressants. The heterogeneity was mild to moderate for these estimates. Approximately half of the included studies reported adverse events. We should note that it is difficult to tell whether the antidepressant therapies, the anticancer therapies or both were responsible for the adverse effects the patients experienced. Adverse events of cardiac toxicity occurred less frequently in the CHM group than in the no treatment group; patients who received CHM treatment also experienced fewer functional gastrointestinal disorders and sleep disturbances and less blurred vision and fatigue than those who received antidepressant treatment. Weight changes and sexual dysfunction, the other common adverse effects of antidepressants, especially of SSRIs [57, 58], were not reported in any of the trials. Withdrawal due to adverse events was reported only in one study. In addition, although CHM seemed to have a beneficial effect on quality of life, that conclusion was not convincing as only three trials explored that outcome.

### Strengths and limitations

This was the first systematic review and meta-analysis to focus on the efficacy and safety of CHM for the treatment of depression or depressive symptoms in cancer patients. During the review process, we tried to prevent and avoid any potential biases. We conducted a comprehensive search of the appropriate databases for published and unpublished trials. Two authors independently searched the literature, selected the studies and extracted the data to reduce potential bias in these complex processes. The included RCTs enrolled participants with different types of cancer, investigated the effectiveness of twelve Chinese herbal preparations for the treatment of depression and depressive symptoms, and compared the CHM intervention to various types of interventions. Although the diversity of characteristics created generally high heterogeneity, it also increased the generalizability of the results. In addition, we included both desirable and undesirable effects of CHM. However, there were some issues that could limit the applicability and reliability of the evidence summarized in this review.

First, all the trials were of poor methodological quality and were inadequately reported. Most of the studies were unable to provide sufficient information, such as the allocation concealment procedure and the dropout rate, which contributed to the unclear risk of selection bias and attrition bias. CHMs always display unique characteristics, particularly when taken in the form of a decoction, making it difficult to produce an appropriate placebo; thus, in our review, all the included trials were considered to have a high risk of bias in the domain of blinding.

Another critical issue that should be considered was the high degree of heterogeneity among the studies, which could not be explained by the diversity in the type of psychiatric diagnosis according to the subgroup analysis. The clinical practice of traditional Chinese medicine (TCM) takes the differentiation of syndromes and treatments as its diagnostic and therapeutic features, resulting in diversity in CHM interventions in terms of ingredients, dosages, and administration. Moreover, participant factors (e.g., age, sex, type of cancer, severity of disease) and study factors (e.g., concordance rates, quality of reporting, outcome assessment tools) were also heterogeneous among the studies. However, such underlying sources of heterogeneity were not confirmed by statistical analyses because no data were available for appropriate comparisons.

Additionally, many trials focused only on outcomes related to depression and ignored the influence of interventions on the quality of life, side effects and dropout rate, which made the evidence for these outcomes less convincing.

Furthermore, all these trials were conducted in China, while CHM is also commonly used in other Asian countries, such as Japan, Vietnam, and Korea; thus, the possibility of potential language bias could not be ruled out.

### Implications for practice

This review revealed that several CHMs could generate beneficial clinical effects and appeared to be safer than conventional treatments for the treatment of depression in patients with cancer. For patients who take antidepressants or undergo psychological treatments, the addition of CHM as an adjuvant therapy is suggested. However, based on the available evidence, CHM cannot replace antidepressants entirely, although CHM is a promising option when patients find the side effects of antidepressants intolerable. Twelve different types of herbal medicines were tested in these trials; Xiao Yao decoction and its modifications were the most frequently used, followed by Banxia Houpo decoction combined with Liu Junzi decoction, Chaihu Shugan decoction, and Ganmai Dazao decoction.

### Suggestions for future research

More rigorous multi-center RCTs with comprehensive and transparent reporting are warranted in the future. Several studies have been published that aimed to improve the quality of RCTs for CHM, and it is suggested that researchers receive systemic training regarding clinical trial design, pre-register their trials with the relevant platforms, collaborate with researchers in different fields and adopt the CONSORT checklist as the reporting quality standard [59–61]. More outcomes should be examined, including quality of life, the effects of overdoses and the use of CHM during pregnancy and lactation. Participants should also be followed for an extended period to assess the long-term effects of the treatment. In addition, it is challenging to summarize the effect of a particular formula because the CHM interventions varied among these trials. Although Xiao Yao decoction seemed to be more effective than the other decoctions tested, further analysis was not conducted due to the limited data. To overcome this problem, clinical trials could enroll patients with specific syndromes and administer standardized CHM interventions.

## Conclusions

According to the investigation of the twelve herbal preparations, the CHM intervention appears to alleviate depressive symptoms for cancer patients, either alone or in combination with antidepressants or psychological treatments. However, a high risk of bias and high heterogeneity made the mean estimates uncertain. Well-designed trials with comprehensive and transparent reporting are warranted in the future. More homogeneous trials are required for a meta-analysis to determine which herbal prescriptions or specific herbs are effective in different combinations.

## Additional files

Additional file 1: Search strategy (DOCX): a text listing the search strategies of CENTRAL, MEDLINE, EMBASE, PsycINFO, CNKI, VIP, SinoMed, and Wanfang Databases. (DOCX 24 kb)

Additional file 2: Content of the twelve Chinese herbal preparations (DOCX): a table listing the content of the twelve Chinese herbal preparations. (DOCX 60 kb)

Additional file 3: Effect estimates summary (DOCX): a table listing the effect estimates of all comparisons and all subgroup-analyses. (DOCX 31 kb)

## Abbreviations

BA: behavioral activation
BDI: Beck Depression Inventory
CBT: cognitive behavioral therapy
CCMD: Chinese Classification of Mental Disorders
CES-D: Center for Epidemiologic Studies–Depression Scale
CHM: Chinese herbal medicine
CI: confidence interval
CNKI: Chinese National Knowledge Infrastructure Databases
DSM: Diagnostic and Statistical Manual of Mental Disorders
EORTC QLQ C-30: European Organization for Research on Treatment of Cancer Quality of Life Questionnaire-30
HADS: Hospital Anxiety and Depression Scale
HAMD: Hamilton Depression Scale
ICD: International Classification of Diseases
ICTRP: International Clinical Trial Registry Platform
IOM: Institute of Medicine
IPT: interpersonal therapy
IT: individualized treatments
MD: mean difference
NCCN: National Comprehensive Cancer Network
NICE: National Institute for Health and Care Excellence
NIH: National Institutes of Health
NIT: non-individualized treatments
NR: not reported
PT: psychological treatments
RCTs: randomized controlled trials
RR: relative risk
SDS: Self-Rating Depression Scale
SinoMed: Chinese Biomedical Database web
SMD: standardized mean difference
TCM: traditional Chinese medicine
VIP: Chinese VIP information
WHO: World Health Organization
