# Supplementary material for: Twelve Chinese herbal preparations for the treatment of depression or depressive symptoms in cancer patients: a systematic review and meta-analysis of randomized controlled trials
Source: BMC Complement Altern Med. 2019 Jan 23;19:28. doi: 10.1186/s12906-019-2441-8 (PMC6345004; doi:10.1186/s12906-019-2441-8)
Supplement: Supplementary file 1 — Search strategy (DOCX): a text listing the search strategies of CENTRAL, MEDLINE, EMBASE, PsycINFO, CNKI, VIP, SinoMed, and Wanfang Databases. (DOCX 24 kb) [file 12906_2019_2441_MOESM1_ESM.docx]

**Search strategy**

**Electronic databases**

1. **CNKI 2017-5-3 (n=451)**

**Strategies in original language:**

SU=('中医药'+'中草药'+'中成药'+'中药'+'传统医药'+'传统医学'+'中西医结合'+'补充替代医学'+'补充替代疗法') AND SU=('肿瘤'+'癌症'+'肉瘤'+'癌'+'恶性'+'白血病'+'淋巴瘤') AND SU=('抑郁症'+'抑郁状态'+'抑郁'+'心境恶劣'+'郁证'+'情绪') AND FT=('随机'+'盲法'+'安慰剂'+'meta分析')

**Strategies in English:**

SU=('traditional Chinese medicine' + 'Chinese herbal medicine' + 'Chinese patent medicine' + ' Chinese medicine' + 'traditional medicine' + 'traditional medicine' + 'Integrative medicine' + 'complementary and alternative medicine' + 'complementary and alternative therapy') AND SU=('tumor' + 'cancer' + 'sarcoma' + 'neoplastic' + 'malignant' + 'leukemia' + 'lymphoma') AND SU=('depression' + 'depressive symptoms' + 'depressed' + 'dysthymia' + 'syndrome of yu' + 'mood') AND FT=('random' + 'blind' + 'placebo' + 'meta-analysis')

1. **Wanfang 2017-5-3 (n=176)**

**Strategies in original language**

主题:(中医药 or 中草药 or 中成药 or 中药 or 传统医药 or 传统医学 or 中西医结合 or 补充替代医学 or 补充替代疗法) * 主题:(肿瘤 or 肉瘤 or 癌 or 恶性 or 白血病 or 淋巴瘤) * 主题:(抑郁症 or 抑郁状态 or 抑郁 or 心境恶劣 or 郁证 or 情绪) * (随机 or 盲法 or 安慰剂 or “meta分析”)

**Strategies in English:**

SU: (traditional Chinese medicine or Chinese herbal medicine or Chinese patent medicine or Chinese medicine or traditional medicine or traditional medicine or Integrative medicine or complementary and alternative medicine or complementary and alternative therapy) * SU: (tumor or cancer or sarcoma or neoplastic or malignant or leukemia or lymphoma) * SU: (depression or depressive symptoms or depressed or dysthymia or syndrome of yu or mood) * (random or blind or placebo or "meta-analysis")

1. **VIP 2017-5-3 (n=24)**

**Strategies in original language**

(M=(中医药+中草药+中成药+中药+传统医药+传统医学+中西医结合+补充替代医学+补充替代疗法))*(M=(肿瘤+肉瘤+癌+恶性+白血病+淋巴瘤))*(M=(抑郁症+抑郁状态+抑郁+心境恶劣+郁证+情绪))*(U=(随机+盲法+安慰剂+meta分析))

**Strategies in English:**

(M=(traditional Chinese medicine + Chinese herbal medicine + Chinese patent medicine + Chinese medicine + traditional medicine + traditional medicine + Integrative medicine + complementary and alternative medicine + complementary and alternative therapy))*(M=(tumor + cancer + sarcoma + neoplastic + malignant + leukemia + lymphoma))*(M=(depression + depressive symptoms + depressed + dysthymia + syndrome of yu + mood))*(U=(random + blind + placebo + meta-analysis)

1. **Sinomed 2017-5-3 (n=203)**

**Strategies in original language**

#1 (((((((("中医药"[常用字段:智能]) OR "中草药"[常用字段:智能]) OR "中成药"[常用字段:智能]) OR "中药"[常用字段:智能]) OR "传统医药"[常用字段:智能]) OR "传统医学"[常用字段:智能]) OR "中西医结合"[常用字段:智能]) OR "补充替代疗法"[常用字段:智能]) OR "补充替代医学"[常用字段:智能]

#2 ((((("肿瘤"[常用字段:智能]) OR "肉瘤"[常用字段:智能]) OR "癌"[常用字段:智能]) OR "恶性"[常用字段:智能]) OR "白血病"[常用字段:智能]) OR "淋巴瘤"[常用字段:智能]

#3 ((((("抑郁症"[常用字段:智能]) OR "抑郁状态"[常用字段:智能]) OR "抑郁"[常用字段:智能]) OR "心境恶劣"[常用字段:智能]) OR "郁证"[常用字段:智能]) OR "情绪"[常用字段:智能]

#4 ((("随机"[全字段:智能]) OR "盲法"[全字段:智能]) OR "安慰剂"[全字段:智能]) OR "meta分析"[全字段:智能]

#5 (#4) AND (#3) AND (#2) AND (#1)

**Strategies in English:**

#1 (((((((("traditional Chinese medicine"[Common fields: intelligence]) OR "Chinese herbal medicine"[Common fields: intelligence]) OR "Chinese patent medicine"[Common fields: intelligence]) OR "Chinese medicine"[Common fields: intelligence]) OR "traditional medicine"[Common fields: intelligence]) OR "traditional medicine"[Common fields: intelligence]) OR "Integrative medicine"[Common fields: intelligence]) OR "complementary and alternative medicine"[Common fields: intelligence]) OR "complementary and alternative therapy"[Common fields: intelligence]

#2 ((((("tumor"[Common fields: intelligence]) OR "cancer"[Common fields: intelligence]) OR "sarcoma"[Common fields: intelligence]) OR "neoplastic"[Common fields: intelligence]) OR "malignant"[Common fields: intelligence]) OR "leukemia"[Common fields: intelligence]) OR "lymphoma"[Common fields: intelligence]

#3 ((((("depression"[Common fields: intelligence]) OR "depressive symptoms"[Common fields: intelligence]) OR "depressed"[Common fields: intelligence]) OR "dysthymia"[Common fields: intelligence]) OR "syndrome of yu"[Common fields: intelligence]) OR "mood"[Common fields: intelligence]

#4 ((("random"[All fields: intelligence]) OR "blind"[All fields: intelligence]) OR "placebo"[All fields: intelligence]) OR "meta-analysis"[All fields: intelligence]

#5 (#4) AND (#3) AND (#2) AND (#1)

**5. Ovid-medline 2017-5-3 (n=99)**

#1 Drugs, Chinese Herbal/

#2 Medicine, Traditional/

#3 Herbal Medicine/

#4 exp Plant Extracts/

#5 exp Phytotherapy/

#6 Plants, Medicinal/

#7 herb or herbs or herba* or plant or plants or traditional medicine* or herbal medicine* or Chinese medicine* or oriental medicine* or phytomedicine or botanical

#8 (#2 OR #3 OR #4 OR #5 OR #6 OR #7)

#9 exp neoplasms/

#10 neoplasm* or cancer or cancers or tumor or tumors or tumour or tumours or carcinoma* or malignan* or metastas* or metastat*

#11 adenocarcinoma* or sarcoma* or lymphoma* or choriocarcinoma* or leukemia* or leukaemia* or teratoma* or melanoma* or blastoma* or glioma* or chordoma* or mesothelioma*

#12 (#9 OR #10 OR #11)

#13 Depression/
#14 exp Depressive Disorder/
#15 Adjustment Disorders/

#16 Mental Health/

#17 depress* or melanchol* or dysthymi* or MDD

#18 (depress* or mood or affectiv* or adjustment or reactive or dysthymi*) adj5 disorder*

#19 (#13 OR #14 OR #15 OR #16 OR #17 OR #18)

#20 random* or blind* or placebo or "meta analysis"

#21 (#8 AND #12 AND #19 AND #20)

**6. Central 2017-5-3 (n=100)**

#1 MeSH descriptor: [herbal medicine] explode all trees

#2 MeSH descriptor: [Medicine, Traditional] explode all trees

#3 MeSH descriptor: [drugs,Chinese herbal] explode all trees

#4 MeSH descriptor: [plants,medicinal] explode all trees

#5 herb or herbs or herba* or plant or plants or traditional medicine* or herbal medicine* or Chinese medicine* or oriental medicine* or phytomedicine or botanical

#6 (#1 OR #2 OR #3 OR #4 OR #5)

#7 MeSH descriptor: [neoplasms] explode all trees

#8 neoplasm* or cancer or cancers or tumor or tumors or tumour or tumours or carcinoma* or malignan* or metastas* or metastat*

#9 adenocarcinoma* or sarcoma* or lymphoma* or choriocarcinoma* or leukemia* or leukaemia* or teratoma* or melanoma* or blastoma* or glioma* or chordoma* or mesothelioma*

#10 (#7 OR #8 OR #9)

#11 MeSH descriptor: [Depression] explode all trees

#12 MeSH descriptor: [Depressive Disorder] explode all trees

#13 MeSH descriptor: [Adjustment Disorders] explode all trees

#14 MeSH descriptor: [Mental Health] explode all trees

#15 depress* or melanchol* or dysthymi* or MDD

#16 (depress* or mood or affectiv* or adjustment or reactive or dysthymi*) NEAR/5 disorder*

#17 (#11 OR #12 OR #13 OR #14 OR #15 OR #16)

#18 (#6 AND #10 AND #17)

**7. Ovid Embase 2017-5-3 (n=1617)**

#1 exp traditional medicine/

#2 Chinese drug/

#3 exp medicinal plant/

#4 exp plant medicinal product/

#5 phytotherapy/

#6 herb or herbs or herba* or plant or plants or traditional medicine* or herbal medicine* or Chinese medicine* or oriental medicine* or phytomedicine or botanical

#7 (#1 OR #2 OR #3 OR #4 OR #5 OR #6)

#8 exp neoplasm/

#9 neoplasm* or cancer or cancers or tumor or tumors or tumour or tumours or carcinoma* or malignan* or metastas* or metastat*

#10 adenocarcinoma* or sarcoma* or lymphoma* or choriocarcinoma* or leukemia* or leukaemia* or teratoma* or melanoma* or blastoma* or glioma* or chordoma* or mesothelioma*

#11 (#8 OR #9 OR #10)

#12 exp depression/
#13 adjustment disorder/

#14 exp mental health/

#15 depress* or melanchol* or dysthymi* or MDD

#16 (depress* or mood or affectiv* or adjustment or reactive or dysthymi*) adj5 disorder*

#17 (#12 OR #13 OR #14 OR #15 OR #16)

#18 random* or blind* or placebo or "meta analysis"

#19 (#7 AND #11 AND #17 AND #18)

**8. PsycINFO 2017-5-3 (n=26)**

#1 exp alternative medicine/

#2 exp “medicinal herbs and plants”

# Chinese cultural groups/

#3 herb or herbs or herba* or plant or plants or traditional medicine* or herbal medicine* or Chinese medicine* or oriental medicine* or phytomedicine or botanical

#4 (#1 OR #2 OR #3)

#5 exp NEOPLASMS/

#6 neoplasm* or cancer or cancers or tumor or tumors or tumour or tumours or carcinoma* or malignan* or metastas* or metastat*

#7 adenocarcinoma* or sarcoma* or lymphoma* or choriocarcinoma* or leukemia* or leukaemia* or teratoma* or melanoma* or blastoma* or glioma* or chordoma* or mesothelioma*

#8 (#5 OR #6 OR #7)

#9 “depression (emotion)”/

#10 exp major depression/

#11 exp mental health/

#12 depress* or melanchol* or dysthymi* or MDD

#13 (depress* or mood or affectiv* or adjustment or reactive or dysthymi*) adj5 disorder*

#14 (#9 OR #10 OR #11 OR #12 OR #13)

#15 random* or blind* or placebo or "meta analysis"

#16 (#4 AND #8 AND #14 AND #15)

**For ongoing trials**

**1. Websites of Chinese clinical trial registry**

http://www.chictr.org.cn/

search"抑郁 (i.e., depression)", 123, no relevant trials（5-5）

**2. International clinical trial registry by U.S. National Institutes of Health**

https://www.clinicaltrials.gov/

search “cancer|interventional studies| depression or depressive symptoms”, 75, one relevant trial

(Clinical trials.gov Identifier: NCT00066859; https://www.clinicaltrials.gov/ct2/show/study/NCT00066859?term=cancer&type=Intr&cond=Depression+or+depressive+symptoms&draw=3&rank=27)

**3. ICTRP**

http://apps.who.int/

search “cancer” and “depression or depressive symptoms”, 40, no relevant trials
