# Supplementary material for: Twelve Chinese herbal preparations for the treatment of depression or depressive symptoms in cancer patients: a systematic review and meta-analysis of randomized controlled trials
Source: BMC Complement Altern Med. 2019 Jan 23;19:28. doi: 10.1186/s12906-019-2441-8 (PMC6345004; doi:10.1186/s12906-019-2441-8)
Supplement: Supplementary file 2 — Content of the twelve Chinese herbal preparations (DOCX): a table listing the content of the twelve Chinese herbal preparations. (DOCX 60 kb) [file 12906_2019_2441_MOESM2_ESM.docx]

TABLE 2：Content of CHM

| **CHM** | **Included studies** | **Comparison** | **Content** |
| --- | --- | --- | --- |
| Banxiahoupo combined with liujunzi decoction | Dai et al. 2017  [28]  Xu et al. 2009  [56] | CHM+ psychological treatment vs.  psychological treatment  CHM+ psychological treatment vs.  psychological treatment | Fuling (Poria), Baizhu (Rhizoma Atractylodis Macrocephalae), Renshen (Radix et Rhizoma Ginseng), Gancao (Radix et Rhizoma Glycyrrhizae), Hongzao (Fructus Jujubae), Suye (Folium Perillae), Houpo (Cortex Magnoliae Officinalis), Chenpi (Pericarpium Citri Reticulatae), Banxia (Rhizoma Pinelliae), Shengjiang (Rhizoma Zingiberis Recens), Lianqiao (Fructus Forsythiae) |
| Modified Xiao Yao decoction | Liu et al. 2011  [27] | CHM vs. antidepressants | Chaihu(Radix Bupleuri), Baishao (Radix Paeoniae Alba),Fuling (Poria), Baizhu (Rhizoma Atractylodis Macrocephalae), Danggui (Radix Angelicae Sinensis), Gancao (Radix et Rhizoma Glycyrrhizae), Xiakucao (Spica Prunellae), Chonglou (Rhizoma Paridis), Shancigu (Pseudobulbus Cremastrae seu Pleiones) |
|  | Jin et al. 2016  [50] | CHM vs. antidepressants | Chaihu(Radix Bupleuri), Baishao (Radix Paeoniae Alba),Fuling (Poria), Baizhu (Rhizoma Atractylodis Macrocephalae), Danggui (Radix Angelicae Sinensis), Gancao (Radix et Rhizoma Glycyrrhizae), Zhiqiao (Fructus Aurantii), Dangshen (Radix Codonopsis), Chuanxiong (Rhizoma Chuanxiong), Xianlingpi (Herba Epimedii), Ezhu (Rhizoma Curcumae), Zhebei (Bulbus Fritillariae Thunbergii), Sheliugu (Amorphophallus rivieri Durieu) |
|  | Ma et al.2005  [43] | CHM vs. no treatment | Chaihu(Radix Bupleuri), Baishao (Radix Paeoniae Alba),Fuling (Poria), Baizhu (Rhizoma Atractylodis Macrocephalae), Danggui (Radix Angelicae Sinensis), Gancao (Radix et Rhizoma Glycyrrhizae), Danpi (Cortex Moutan), Zhizi (Fructus Gardeniae), Bohe (Herba Menthae), Danshen (Radix et Rhizoma Salviae Miltiorrhizae), Banzhilian (Herba Scutellariae Barbatae), Hehuanpi (Cortex Albiziae) |
|  | Sun et al.2016  [46] | CHM vs. no treatment | Chaihu(Radix Bupleuri), Baishao (Radix Paeoniae Alba),Fuling (Poria), Baizhu (Rhizoma Atractylodis Macrocephalae), Danggui (Radix Angelicae Sinensis), Gancao (Radix et Rhizoma Glycyrrhizae), Bohe (Herba Menthae), Shengjiang (Rhizoma Zingiberis Recens), Dazao (Rhizoma Zingiberis Recens), Shudihuang (Radix Rehmanniae Praeparata), Nvzhenzi (Fructus Ligustri Lucidi), Mohanlian (Herba Ecliptae), Zexie (Rhizoma Alismatis) |
| Modified Ganmai Dazao decoction | Meng et al. 2017 [45]  Zhang et al. 2015  [53] | CHM vs. no treatment  CHM vs. antidepressants | Chaihu(Radix Bupleuri), Baishao (Radix Paeoniae Alba),Fuling (Poria), Baizhu (Rhizoma Atractylodis Macrocephalae), Danggui (Radix Angelicae Sinensis), Gancao (Radix et Rhizoma Glycyrrhizae), Dazao (Rhizoma Zingiberis Recens), Zhiqiao (Fructus Aurantii), Hehuanpi (Cortex Albiziae), Yujin (Radix Curcumae), Fuxiaomai (Fructus Tritici Levis), Suanzaoren (Semen Ziziphi Spinosae) |
| Chaihu Shugan San decoction | Fang et al. 2013  [48]  Zheng et al. 2002  [54] | CHM vs. antidepressants  CHM vs. psychological treatment | Chaihu(Radix Bupleuri), Gancao (Radix et Rhizoma Glycyrrhizae), Huangqin (Radix Scutellariae), Yujin (Radix Curcumae), Shichangpu (Rhizoma Acori Tatarinowii), Zhishi (Fructus Aurantii Immaturus),Taoren (Semen Persicae), Honghua (Flos Carthami), Muxiang (Radix Aucklandiae), Chenpi (Pericarpium Citri Reticulatae), Baiziren (Semen Platycladi), Shenglonggu (Os Draconis), Shengmuli (Concha Ostreae), Yuanzhi (Radix Polygalae), Danshen (Radix et Rhizoma Salviae Miltiorrhizae), Banxia (Rhizoma Pinelliae), Houpo (Cortex Magnoliae Officinalis) |
| Chaihu Shugan San decoction and Tongyou decoction alternatively | Meng et al. 1999  [44] | CHM vs. no treatment | Chaihu Shugan San decoction  Chaihu(Radix Bupleuri), Baishao (Radix Paeoniae Alba), Gancao (Radix et Rhizoma Glycyrrhizae), Chuanxiong (Rhizoma Chuanxiong), Chenpi (Pericarpium Citri Reticulatae), Zhiqiao (Fructus Aurantii), Xiangfu (Rhizoma Cyperi)  Tongyou decoction  Danggui (Radix Angelicae Sinensis), Gancao (Radix et Rhizoma Glycyrrhizae), Taoren (Semen Persicae), Honghua (Flos Carthami), Shengdihuang (Radix Rehmanniae), Shudihuang (Radix Rehmanniae Praeparata), Shengma (Rhizoma Cimicifugae) |
| Chaihu jia longgumuli decoction | Tian et al. 2012  [52] | CHM vs. antidepressants | Chaihu(Radix Bupleuri), Shengjiang (Rhizoma Zingiberis Recens), Dazao (Rhizoma Zingiberis Recens), Fuling (Poria), Cishi (Magnetitum), Dahuang (Radix et Rhizoma Rhei), Zhizi (Fructus Gardeniae), Lianqiao (Fructus Forsythiae), Chenpi (Pericarpium Citri Reticulatae), Dangshen (Radix Codonopsis), Huangqin (Radix Scutellariae), Banxia (Rhizoma Pinelliae), Guizjhi (Ramulus Cinnamomi) |
| Modified Xuefuzhuyu decoction | Chen et al. 2016  [26] | CHM vs. no treatment | Chaihu(Radix Bupleuri), Danggui (Radix Angelicae Sinensis), Gancao (Radix et Rhizoma Glycyrrhizae), Taoren (Semen Persicae), Honghua (Flos Carthami), Shengdihuang (Radix Rehmanniae), Zhiqiao (Fructus Aurantii), Chishao (Radix Paeoniae Rubra), Jiegeng (Radix Platycodonis), Niuxi(Radix Achyranthis Bidentatae) |
| Suanzaoren jia longmu decoction | Fu et al. 2012  [49] | CHM vs. antidepressants | Fuling (Poria), Gancao (Radix et Rhizoma Glycyrrhizae), Suanzaoren (Semen Ziziphi Spinosae), Chuanxiong (Rhizoma Chuanxiong), Zhimu (Rhizoma Anemarrhenae), Shenglonggu (Os Draconis), Shengmuli (Concha Ostreae) |
| self-formulated decoction | Jia et al. 2016  [55] | CHM+ antidepressants vs. antidepressants | Chaihu (Radix Bupleuri), Fuling (Poria), Baizhu (Rhizoma Atractylodis Macrocephalae), Shenglonggu (Os Draconis), Shengmuli (Concha Ostreae), Yujin (Radix Curcumae), Chenpi (Pericarpium Citri Reticulatae), Shichangpu (Rhizoma Acori Tatarinowii), Ezhu (Rhizoma Curcumae), Gegen (Radix Puerariae Lobatae), Taizishen(Radix Pseudostellariae), Suanzaoren (Semen Ziziphi Spinosae), Baihuasheshecao (Herba Hedyotis Diffusae), Beimu (Bulbus Fritillaria), Yuanzhi (Radix Polygalae) |
| Yangfei xiaojijieyu decoction | Liu et al. 2015  [42] | CHM vs. no treatment | Baizhu (Rhizoma Atractylodis Macrocephalae), Cangzhu (Rhizoma Atractylodis), Nanshashen (Radix Adenophorae), Beishashen (Radix Glehniae), Huangqi (Radix Astragali), Fangfeng (Radix Saposhnikoviae), Yiyiren (Semen Coicis), Xiakucao (Spica Prunellae), Shishangbai (Herba Selaginellae Doederleinii), Quanxie (Scorpio), Yujin (Radix Curcumae), Meiguihua (Flos Rosae Rugosae), Meihua (Flos Mume), Hehuanpi (Cortex Albiziae), Yejiaoteng (Caulis Polygoni Multiflori) |
| Shugan jieyuhuaji decoction | Ma et al. 2009  [51] | CHM vs. antidepressants | Chaihu (Radix Bupleuri), Baizhu (Chaihu (Radix Bupleuri), Baishao (Radix Paeoniae Alba), Gancao (Radix et Rhizoma Glycyrrhizae), Chenpi (Pericarpium Citri Reticulatae), Banxia (Rhizoma Pinelliae), Hehuanpi (Cortex Albiziae), Baihe (Bulbus Lilii), Sheshecao (Herba Hedyotis Diffusae), Maozhuacao (Radix Ranunculi Ternati), Zhiqiao (Fructus Aurantii) |
| Shugan Jieyu capsule | Wu et al. 2014  [47] | CHM vs. no treatment | Guanyejinsitao (Herba Hyperici Perforati), Ciwujia (Radix et Rhizoma seu Caulis Acanthopanacis Senticosi) |

CHM: Chinses herbal medicine
