# Supplementary material for: Twelve Chinese herbal preparations for the treatment of depression or depressive symptoms in cancer patients: a systematic review and meta-analysis of randomized controlled trials
Source: BMC Complement Altern Med. 2019 Jan 23;19:28. doi: 10.1186/s12906-019-2441-8 (PMC6345004; doi:10.1186/s12906-019-2441-8)
Supplement: Supplementary file 3 — Effect estimates summary (DOCX): a table listing the effect estimates of all comparisons and all subgroup-analyses. (DOCX 31 kb) [file 12906_2019_2441_MOESM3_ESM.docx]

| **Outcome or subgroup** | **No. of trials** | **Effect estimates [95%CI]** | **P value** | **reference** |
| --- | --- | --- | --- | --- |
| **1. depression: group mean scores SMD [95%CI]** | |  | |  |
| **1.1. CHM vs. no treatment** | |  | |  |
| subgroup of patients with depression | 5 | -1.79 [-3.16, -0.41] | 0.01 | [26,42,43,45,46] |
| subgroup of patients with depressive symptoms | 2 | -3.61 [-6.46, -0.75] | 0.01 | [44, 47] |
| **Meta-analysis (heterogeneity: I^2^ = 97%, P<0.00001)** | 7 | -2.30 [-3.54,-1.05] | 0.0003 | [26, 42-47] |
| **Subgroup differences (heterogeneity: I^2^ = 21.3%, P=0.26)** | |  | |  |
| **1.2. CHM vs. antidepressants** | |  | |  |
| subgroup of patients with depression | 6 | -0.57 [-1.07, -0.06] | 0.03 | [27, 49-52] |
| subgroup of patients with depressive symptoms | 1 | -0.80 [-1.23, -0.37] | 0.0003 | [48] |
| **Meta-analysis (heterogeneity: I^2^ = 82%, P<0.0001)** | 7 | -0.61 [-1.03,-0.18] | 0.005 | [27, 48-52] |
| **Subgroup differences (heterogeneity: I^2^ = 0%, P=0.49)** | |  | |  |
| **1.3. CHM + psychological treatments vs. psychological treatments** | |  | |  |
| patients with depression | 1 | -0.55 [-1.07,-0.02] | 0.04 | [56] |
| **2. depression: treatment response rate RR [95%CI]** | |  |  |  |
| **2.1. CHM vs. no treatment** |  |  |  |  |
| subgroup of patients with depression | 3 | 1.65 [0.92, 2.97] | 0.09 | [26, 42, 43] |
| subgroup of patients with depressive symptoms | 1 | 1.83 [1.25, 2.69] | 0.002 | [47] |
| **Meta-analysis (heterogeneity: I^2^ = 33%, P=0.21)** | 4 | 1.65 [1.19, 2.29] | 0.003 | [26, 42, 43, 47] |
| **Subgroup differences (heterogeneity: I^2^ = 0%, P=0.77)** | |  | |  |
| **2.2. CHM vs. antidepressants** |  |  |  |  |
| subgroup of patients with depression | 5 | 1.04 [0.90, 1.20] | 0.58 | [27, 49 ,51-53] |
| subgroup of patients with depressive symptoms | 1 | 1.35 [0.90, 2.02] | 0.15 | [48] |
| **Meta-analysis (heterogeneity: I^2^ = 18%, P=0.30)** | 6 | 1.08 [0.93, 1.26] | 0.31 | [27,48,49,51-53] |
| **Subgroup differences (heterogeneity: I^2^ =29.7%, P=0.23)** | |  | |  |
| **2.3. CHM vs. psychological treatments** |  |  |  |  |
| patients with depression | 1 | 1.15 [1.03, 1.28] | 0.01 | [54] |
| **2.4. CHM + antidepressants vs. antidepressants** | |  | |  |
| patients with depression | 1 | 1.32 [1.07, 1.63] | 0.009 | [55] |
| **2.5. CHM + psychological treatments vs. psychological treatments** | |  | |  |
| patients with depression | 2 | 1.70 [1.02, 2.85] | 0.04 | [28, 56] |
| **Meta-analysis (heterogeneity: I^2^ = 61%, P=0.11)** | 2 | 1.70 [1.02, 2.85] | 0.04 | [28, 56] |
| **3. Quality of life: group mean scores SMD [95%CI]** | |  | |  |
| **3.1. CHM vs. no treatment** |  |  |  |  |
| patients with depressive symptoms | 1 | 13.70 [10.08, 17.32] | <0.00001 | [45] |
| **3.2. CHM vs. antidepressants** |  |  |  |  |
| patients with depression | 1 | -0.37 [-0.88, 0.14] | 0.16 | [51] |
| **4. Quality of life: treatment response rate RR [95%CI]** | |  |  |  |
| **4.1. CHM vs. no treatment** |  |  |  |  |
| patients with depression | 2 | 1.60 [1.17, 2.18] | 0.003 | [42, 46] |
| **Meta-analysis (heterogeneity: I^2^ = 0%, P=0.84)** | 2 | 1.60 [1.17, 2.18] | 0.003 | [42, 46] |
| **5. Adverse events RR [95%CI]** | |  |  |  |
| **5.1. CHM vs. no treatment** |  |  |  |  |
| **5.1.1. functional gastrointestinal disorders** |  |  |  |  |
| patients with depression | 1 | 0.56 [0.31, 1.01] | 0.05 | [46] |
| **5.1.2. leucopenia** |  |  |  |  |
| patients with depression | 1 | 0.53 [0.28, 1.01] | 0.05 | [46] |
| **5.1.3. cardiac toxicity** |  |  |  |  |
| patients with depression | 1 | 0.25 [0.08, 0.80] | 0.02 | [46] |
| **5.1.4. alopecia** |  |  |  |  |
| patients with depression | 1 | 0.82 [0.64, 1.06] | 0.13 | [46] |
| **5.1.5. liver or renal dysfunction** |  |  |  |  |
| patients with depression | 1 | 0.75 [0.29, 1.92] | 0.55 | [46] |
| **5.2. CHM vs. antidepressants** |  |  |  |  |
| **5.2.1. functional gastrointestinal disorders** |  |  |  |  |
| subgroup of patients with depression | 5 | 0.22 [0.06, 0.89] | 0.03 | [27,49,50,52,53] |
| subgroup of patients with depressive symptoms | 1 | 0.22 [0.05, 0.97] | 0.05 | [48] |
| **Meta-analysis (heterogeneity: I^2^ = 46%, P=0.10)** | 6 | 0.24 [0.08, 0.69] | 0.008 | [27,48-50,52,53] |
| **Subgroup differences (heterogeneity: I^2^ =0%, P=0.99**) | |  | |  |
| **5.2.2. headache or dizziness** |  |  |  |  |
| subgroup of patients with depression | 4 | 0.46 [0.17, 1.23] | 0.12 | [27,49,52,53] |
| subgroup of patients with depressive symptoms | 1 | 0.67 [0.12, 3.80] | 0.65 | [48] |
| **Meta-analysis (heterogeneity: I^2^ = 0%, P=0.78)** | 5 | 0.50 [0.21, 1.19] | 0.12 | [27,48,49,52,53] |
| **Subgroup differences (heterogeneity: I^2^ =0%, P=0.72**) | |  | |  |
| **5.2.3. sleep disturbances** |  |  |  |  |
| subgroup of patients with depression | 3 | 0.44 [0.20, 0.99] | 0.05 | [27, 49, 50] |
| subgroup of patients with depressive symptoms | 1 | 0.25 [0.03, 2.15] | 0.21 | [48] |
| **Meta-analysis (heterogeneity: I^2^ = 0%, P=0.66)** | 4 | 0.41 [0.19, 0.88] | 0.02 | [27, 48-50] |
| **Subgroup differences (heterogeneity: I^2^ =0%, P=0.63**) | |  | |  |
| **5.2.4. dry mouth** |  |  |  |  |
| subgroup of patients with depression | 3 | 0.22 [0.04, 1.22] | 0.08 | [27, 49, 53] |
| subgroup of patients with depressive symptoms | 1 | 1.50 [0.26, 8.55] | 0.65 | [48] |
| **Meta-analysis (heterogeneity: I^2^ = 63%, P=0.04)** | 4 | 0.36 [0.08, 1.62] | 0.18 | [27, 48, 49, 53] |
| **Subgroup differences (heterogeneity: I^2^ =58.1%, P=0.12**) | |  | |  |
| **5.2.5. blurred vision** |  |  |  |  |
| subgroup of patients with depression | 1 | 0.04 [0.00, 0.61] | 0.02 | [53] |
| subgroup of patients with depressive symptoms | 1 | 0.20 [0.01, 4.05] | 0.29 | [48] |
| **Meta-analysis (heterogeneity: I^2^ = 0%, P=0.40)** | 2 | 0.08 [0.01, 0.63] | 0.02 | [48, 53] |
| **Subgroup differences (heterogeneity: I^2^ =0%, P=0.42**) | |  | |  |
| **5.2.6. fatigue** |  |  |  |  |
| patients with depression | 1 | 0.20 [0.05, 0.86] | 0.03 | [50] |
| **5.2.7. sweating** |  |  |  |  |
| patients with depression | 1 | 1.00 [0.16, 6.07] | 1.00 | [49] |
| **5.2.8. tachycardia** |  |  |  |  |
| patients with depression | 1 | 0.07 [0.00, 1.14] | 0.06 | [53] |
